# Enhancing automated lower limb rehabilitation exercise task recognition through multi-sensor data fusion in tele-rehabilitation

**DOI:** 10.1186/s12938-024-01228-w

**Published:** 2024-03-19

**Authors:** Alireza Ettefagh, Atena Roshan Fekr

**Affiliations:** 1 KITE Research Institute, Toronto Rehabilitation Institute, University Health Network, 550 University Ave., Toronto, M5G 2A2 Ontario Canada; 2 Institute of Biomedical Engineering, University of Toronto, 164 College St., Toronto, M5S 3E2 Ontario Canada

**Keywords:** Tele-rehabilitation, Exercise recognition, Classification, Data fusion, Convolutional neural network, Deep learning

## Abstract

**Background:**

Tele-rehabilitation is the provision of physiotherapy services to individuals in their own homes. Activity recognition plays a crucial role in the realm of automatic tele-rehabilitation. By assessing patient movements, identifying exercises, and providing feedback, these platforms can offer insightful information to clinicians, thereby facilitating an improved plan of care. This study introduces a novel deep learning approach aimed at identifying lower limb rehabilitation exercises. This is achieved through the integration of depth data and pressure heatmaps. We hypothesized that combining pressure heatmaps and depth data could improve the model’s overall performance.

**Methods:**

In this study, depth videos and body pressure data from an accessible online dataset were used. This dataset comprises data from 30 healthy individuals performing 7 lower limb rehabilitation exercises. To accomplish the classification task, three deep learning models were developed, all based on an established 3D-CNN architecture. The models were designed to classify the depth videos, sequences of pressure data frames, and combination of depth videos and pressure frames. The models’ performance was assessed through leave-one-subject-out and leave-multiple-subjects-out cross-validation methods. Performance metrics, including accuracy, precision, recall, and F1 score, were reported for each model.

**Results:**

Our findings indicated that the model trained on the fusion of depth and pressure data showed the highest and most stable performance when compared with models using individual modality inputs. This model could effectively identify the exercises with an accuracy of 95.71%, precision of 95.83%, recall of 95.71%, and an F1 score of 95.74%.

**Conclusion:**

Our results highlight the impact of data fusion for accurately classifying lower limb rehabilitation exercises. We showed that our model could capture different aspects of exercise movements using the visual and weight distribution data from the depth camera and pressure mat, respectively. This integration of data provides a better representation of exercise patterns, leading to higher classification performance. Notably, our results indicate the potential application of this model in automatic tele-rehabilitation platforms.

## Introduction

### Background

Regular rehabilitation services are essential for patients who suffer from musculoskeletal disorders (MSDs). MSDs encompass a wide range of conditions that can cause chronic pain, mobility impairment, falls, and a decreased quality of life. These disorders primarily affect the muscles, tendons, nerves, ligaments, and other tissues of the body, often leading to inflammation, pain, discomfort, or tingling sensations. Among the various types of MSDs, Lower Limb Disorders (LLDs) specifically target different regions of the lower body, including the hip, thigh, knee, calf, ankle, and foot [1]. These LLDs negatively impact an individual’s ability to move and perform activities of daily living.

Following cancer and cardiovascular diseases, MSD stands as the third leading cause of disease burden in Canada [2]. According to [2], the all-age prevalence of various musculoskeletal conditions increased from 23% in 1990 to 27.8% in 2017. As a result, in 2017, Canada ranked among the top 10 countries globally for the prevalence of several MSDs, such as osteoarthritis and gout. Regular exercise in rehabilitation programs plays a vital role in the management of musculoskeletal conditions [3–6]. This highlights the need for automatic rehabilitation solutions to address the consequences of this growing issue.

Tele-rehabilitation (tele-rehab) is the delivery of medical or rehabilitative services to patients using tele-communication or the internet [7]. Tele-rehab tools reduce distance barriers for patients and researchers, enabling improved access and opening avenues for optimizing intervention strategies in healthcare [8]. In the 1990s, e-health and tele-rehab gained prominence due to advancements in technology [9–11]. In 1998, the U.S. Department of Education’s National Institute for Disability and Rehabilitation Research (NIDRR) initiated the first Rehabilitation Engineering Research Center (RERC) on tele-rehab [12]. This landmark funding aimed to bridge a service delivery gap resulting from managed-care policies limiting the duration of inpatient rehabilitation [8].

Despite the existence of tele-rehab for several decades, its adoption in clinical practice has been limited due to various factors. These include concerns regarding the costs, complexity of implementation, low accuracy, and high incidence of false alarms [8, 13]. These challenges have inhibited the widespread use of tele-rehab solutions and have prevented their full potential from being realized in healthcare settings [8, 13–16].

As defined by RERC on tele-rehab, there is a need for development and evaluation of technologies for assessment and monitoring of progress and outcome of rehabilitation at a distance [13, 17]. One important component of exercise monitoring in an automatic tele-rehab platform is activity recognition, which refers to the process of automatically identifying human activities based on sensor data or visual inputs. In essence, analyzing and understanding movements performed by individuals during their rehabilitation therapy offer valuable insights to clinicians for developing their care plans. These platforms should have the capability to recognize and evaluate different exercises. This is only possible when the computer vision task of activity recognition is accurate, enabling the delivery of meaningful feedback to the users. Consequently, this could potentially assist patients in refining their movements and optimizing their plan of care. To address the exercise recognition problem, this paper proposes a novel deep learning approach that uses an available online dataset to classify different lower limb rehabilitation exercises using privacy-preserving depth information and pressure data. Notably, our approach outperforms the state-of-the-art performance on this dataset.

### Related work

Several studies have also employed different machine learning techniques to perform exercise recognition based on various input data. For instance, Anton *et al.* developed a system using Kinect technology to monitor and evaluate the type and quality of physical rehabilitation exercises in real time [18]. Their system employed two methods: posture classification and exercise recognition. By capturing the spatial coordinates of body joints, the algorithm calculated relative positions, joint angles, and limb angles. These measurements were used to create a posture descriptor consisting of 30 features. Posture classification was performed by comparing the captured descriptor with prestored posture descriptors using Dynamic Time Warping (DTW). For exercise recognition, the system identified the starting and ending postures of each exercise and utilized DTW-based trajectory recognition to assess the accuracy of movement patterns. The proposed algorithm was evaluated through clinical trials involving 15 patients with shoulder disorders. They obtained an accuracy of 95.16% in recognizing 4 different shoulder exercises.

Barriga *et al.* introduced a vision-based system for telecare and tele-rehab using a depth camera and neural networks [19]. They claimed that their system has the capability to automatically classify 7 static postures and falls. The system’s performance was validated using data collected from 6 participants. The researchers also investigated various parameters, including the number of hidden neurons, maximum error, learning rate, and learning function, in the design of their neural network. Additionally, they explored the impact of distance from the camera and the angle between the camera and subjects in the skeleton tracking system. Through their experiments, they achieved an accuracy of 96% for classifying static postures and detecting falls.

Decroos *et al.* developed a machine learning pipeline using Kinect to monitor and assess the correctness of physiotherapy exercises performed by patients at home [20]. Their pipeline involved three main steps: identifying individual exercise repetitions, representing time-series data with statistical features about joint angles, and detecting the exercise’s type, correctness, and possible mistakes. To evaluate the performance of their method, they recorded 10 healthy participants performing 3 rehab exercises (squats, forward lunges, and side lunges) while tracking joint movements with Kinect. For exercise recognition, they used 5 learners, including Linear Regression, Naïve Bayes, Decision Tree, Random Forest, and XGBoost. The input feature vector to the learners consisted of 150 summary statistics (30 joint angles $\times$ 5 statistics - min, max, mean, median, std) for each exercise repetition. The best accuracy achieved was 99% using XGBoost algorithm with Leave-One-Subject-Out (LOSO) cross-validation.

Bijalwan *et al.* proposed a heterogeneous deep learning model to identify lower limb rehabilitation exercises [21]. To this end, they considered a total of 10 exercises involving abduction, flexion, rotation, and dorsi-flexion of the lower limb on both the left and right sides. These exercises were performed by 25 healthy and 10 crouch walking subjects. Depth data were collected from a Kinect v2 sensor. To classify the exercises, they employed Convolutional Neural Network (CNN) and CNN-LSTM models, where LSTM, short for Long Short-Term Memory, is a type of recurrent neural network architecture known for its ability to retain long-term dependencies in data sequences [22]. For validation, a hold-out validation approach was employed, with the dataset split into 50% for training, 20% for validation, and 30% for testing. Their experimental results demonstrated both accuracies and F1 scores of 96% for the CNN model and 98% for the CNN-LSTM model.

Barzegar Khanghah *et al.* proposed a vision-based system to assess the quality of rehabilitation exercises [23]. They used an open dataset consisting of 16 patients and 14 healthy participants performing 9 different rehabilitation exercises. Data were depth videos recorded from a Kinect 1 sensor. They used a pretrained 3D convolutional neural network to perform exercise recognition on correctly executed data as a part of their assessment system. They obtained average accuracies of 96.62% and 86.04% in identifying the exercises using tenfold and LOSO cross validations, respectively.

Wijekoon *et al.* introduced the Multi-modal Exercises Dataset (MEx) as a multi-sensor Human Activity Recognition (HAR) dataset [24]. The data collection involved a pressure mat and a depth camera, both operating at 15 Hz, and two accelerometers operating at 100 Hz. One accelerometer was positioned on the thigh, while the other was placed on the wrist. The dataset includes 7 lower limb exercises performed by 30 healthy participants. Through Leave-Multiple-Subjects-Out (LMSO) cross-validation, the average F1 scores for exercise recognition were 86.34%, 88.92%, 64.99%, and 71.95% using depth data, thigh accelerometer data, wrist accelerometer data, and pressure data, respectively. This study concluded that vision data such as depth provided better results than the time-series data from accelerometers. In subsequent work, the authors proposed a multi-modal Hybrid Attention Fusion (mHAF) deep learning architecture [25]. With a combination of pressure mat, depth camera, and thigh accelerometer data, they achieved an F1 score of 96.24% for exercise recognition using LOSO cross-validation. When pressure and depth data were used without the accelerometer data, the performance was reduced to 90.41%.

Wearable technology shows great potential for lower limb tele-rehab systems. For example, Lai *et al.* achieved 99% accuracy in recognizing 6 lower limb exercises using one Inertial Measurement Unit (IMU). The IMU was attached to the knee for 4 exercises and instep for the other two [26]. García-de-Villa *et al.* classified 8 exercises (5 lower limbs) with 96.2% accuracy [27]. Kim *et al.* also detected Sarcopenia patients with 95% accuracy using IMUs mounted on the left and right feet [28]. Albeit useful, using wearables would be challenging for seniors. One primary obstacle in using wearable technology for seniors is the difficulty they may face in accurately positioning the sensors on their body. They may require external help to properly place the sensors at the appropriate location, angle, and direction. Additionally, research suggests that many older adults are not keen on using such technology. They prefer their usual routines without electronic devices [29]. As a result, they might hesitate to wear sensors on their bodies.

Vision-based technology as a contactless approach offers a great alternative to wearables. These systems often use skeleton tracking models to locate body joints. Such models require Red-Green-Blue (RGB) data to capture body limb movements, which involves recording images or videos of users. This raises potential privacy concerns, as it involves capturing and processing visual information of individuals within their private living spaces. Patients may feel uncomfortable knowing that their movements and activities are being monitored through RGB cameras. This may lead to potential reluctance in using such technologies [30, 31]. Researchers have used depth cameras to mitigate this challenge [32]. The depth data captures only an outline of the body, ensuring complete anonymity. One challenge with vision-based systems is occlusion, where the joints and body parts are hidden from the camera [33]. This is even more likely to happen for exercises that should be performed in lying down positions, i.e., lower limb exercises. The presence of occlusion can negatively impact the performance of exercise recognition models, leading to a decrease in accuracy and reliability.

Given the potential challenges discussed above, we aim to fill these gaps using the fusion of depth and pressure heatmaps. Depth data can provide information about the pattern of body movements without the need for intrusive RGB visuals. Additionally, pressure data can offer insights into the patterns of body limbs and the force exerted on the ground by them during exercises. Our hypothesis is that the combination of pressure distribution data and depth data can enhance a deep learning model’s ability to differentiate between various types of exercises. By leveraging these alternative data sources, we strive to create a more user-friendly and privacy-conscious approach for exercise recognition in lower limb tele-rehab.

## Evaluation

### Methodology

The models were validated using two cross-validation techniques: LOSO and LMSO with 6 groups of 5 individuals. LOSO cross-validation mimics the practical situation where our models encounter new individuals, one at a time, during its application. In addition, the LMSO cross-validation goes beyond LOSO by simulating scenarios where the model is exposed to completely new groups of subjects. To determine the optimal training hyperparameters, including batch size, learning rate, and number of epochs, we employed 5-fold cross-validation with grid search. The performance of the models was evaluated using Eq. (1, 2, 3) as follows:

1 $$\begin{aligned} Accuracy = \frac{TP + TN}{TP + TN + FP + FN} \end{aligned}$$

2 $$\begin{aligned} Precision = \frac{TP}{TP + FP}&\quad , \quad Recall = \frac{TP}{TP + FN}. \end{aligned}$$

The macro F1 score is computed by taking the average of the F1 scores for each exercise. The F1 score for each exercise is determined by calculating the harmonic mean of precision and recall:

3 $$\begin{aligned} F1 Score = 2\times \frac{Precision\times Recall}{Precision+Recall}, \end{aligned}$$

where *TP*, *TN*, *FP*, and *FN* are the number of true positives, true negatives, false positives, and false negatives in the classification of each exercise, respectively.

## Results and discussion

### Classification performance

Among all models, a batch size of 4 yielded the best results. The learning rate was set to 5*e*-5 for the models trained with depth videos (DC) and pressure data (PM), and 1*e*-4 for the model trained with concatenated inputs (DC-PM). For the DC and PM models, the best epoch size was found to be 76, while this was 60 for the DC-PM model. These hyperparameter settings were found to be optimal for both LMSO and LOSO cross-validations. Table 1 presents the classification performance of all three models with both LOSO and LMSO.

**Table 1:** Classification performance for each model

Model	LOSO			
	Accuracy^a^ (%)	Precision (%)	Recall (%)	F1 Score (%)
DC	93.81 ± 7.98	93.85 ± 8.45	93.81 ± 8.48	93.8 ± 7.7
PM	81.43 ± 16.14	81.75 ± 8.56	81.43 ± 8.36	81.45 ± 7.12
DC-PM	95.71 ± 7.51	95.83 ± 6.32	95.71 ± 5.35	95.74 ± 5.19

	LMSO			
	Accuracy (%)	Precision (%)	Recall (%)	F1 Score (%)
DC	90.95 ± 4.49	91.35 ± 8.77	90.95 ± 11.17	90.83 ± 8.28
PM	75.71 ± 5.89	75.48 ± 8.58	75.71 ± 14.49	75.28 ± 10
DC-PM	94.76 ± 1.96	94.84 ± 6.72	94.76 ± 6.34	94.77 ± 5.84

^a^Standard deviations of accuracies are between-subject, and between-class for other metrics

This table shows that the PM model consistently provides the lowest performance among the other models. When considering classification accuracy, there is a relatively large variance across different subjects. This difference might arise from the pressure mat’s capability to capture individual characteristics, such as weight distribution and body shape [24]. This model identifies exercises by analyzing the pattern of body parts in contact with the ground and the force applied to the ground by the active limbs. It uses pressure patterns to determine the exercise type, focusing on how the body engages with the ground during the movement.

While pressure data could be indicative of exercise type based on pressure patterns, they do not capture the same level of detailed information about body movements as the depth camera. As shown in Figures 5 and 6, depth data provide a better view of the body during exercise and capture the entire movement sequence. It includes information about all body parts and their positions relative to the camera. As shown in Table 1, the DC model could better classify the 7 types of exercises with approximately 94% accuracy.

The DC-PM model, which combines both depth camera and pressure mat data, was the most accurate model in identifying the exercises in LOSO. In LMSO, all models experience a decrease in performance compared to LOSO, which is expected due to the reduced subject-specific data for training. Despite the drop in performance, the DC-PM model still provided the highest performance among the other two models. The DC-PM model also demonstrates the most consistent outcomes, as indicated by its low standard deviation in Table 1. This improvement in performance can be attributed to the complementary nature of the two data modalities and how they, as a group, address the limitations of the individual models. More specifically, the combination of visual and weight distribution information from the depth camera and pressure mat allows the model to capture different aspects of exercise movements. This fusion of data provides a richer representation of exercise patterns, leading to higher classification performance.

### Confusion matrices, misclassification charts, and F1 scores

**Fig. 1:**
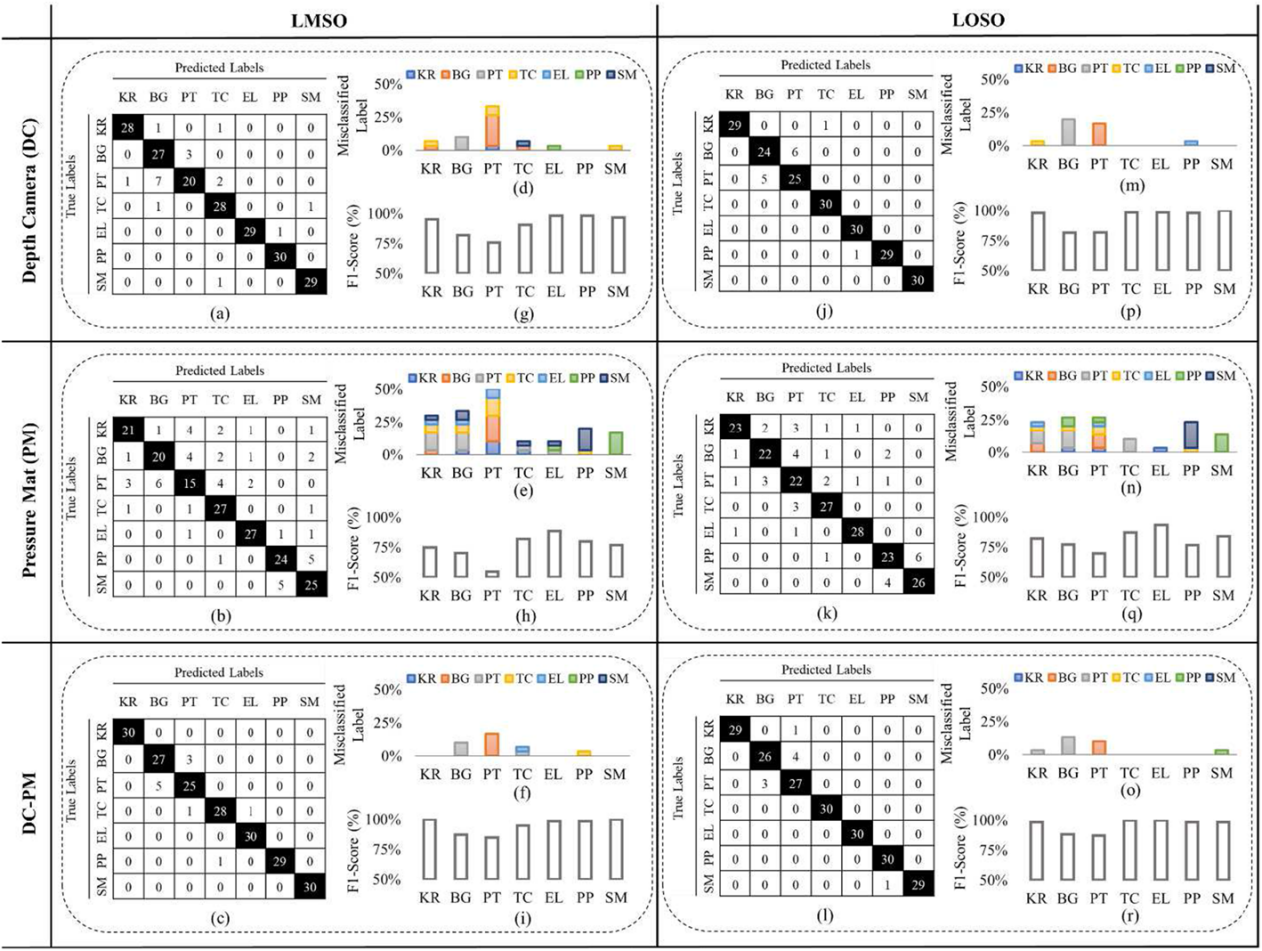
**a**–**c** Confusion matrices, **d**–**f** the proportion of misclassified labels, and **g**–**i** f1 score per exercise for the DC, PM and DC-PM models, respectively, considering the LMSO technique. **j**–**l** Confusion matrices, **m**–**o** the proportion of misclassified labels, and **p**–**r** f1 score per exercise for the DC, PM and DC-PM models, respectively, considering the LOSO technique

Figure 1 (a-c, j-l) presents the confusion matrices for the DC, PM, and DC-PM models, respectively. The misclassified data by each model can be found in Figure 1 (d-f, m-o). The F1 scores for all exercises are displayed in Figure 1 (g-i, p-r).

In most cases, the Bridging (BG) and Pelvic Tilt (PT) exercises were misclassified by each other. This is likely due to their similar starting positions and body trajectories, as evident in Figures 5 and 6. Additionally, the Prone Punches (PP) and Superman (SM) exercises were almost perfectly classified when using depth data; however, considering the pressure data, they were misclassified by the other. Looking at Figures 5 and 6, it is evident that the Repeated Extension in Lying (EL) exercise has distinctive patterns in both depth and pressure data. This exercise, thus, had the lowest misclassification rate when considering the DC and PM models individually.

### Gradient-weighted class activation mapping

To further analyze the results, we used the Gradient-weighted Class Activation Mapping (Grad-CAM) [34] as a technique to visualize and understand the decision-making process of deep learning models. This technique uses the gradients of the classification score flowing into the final convolutional layer of the network to construct a heatmap highlighting the regions of the input that most impact the model’s prediction [34, 35].

To achieve this, we started by inputting a sample into the model, generating both the feature map of the final convolutional layer and the corresponding output prediction. Subsequently, we computed the gradient of the top predicted class with respect to the feature map of the last convolutional layer. This gradient has the same dimensions as the feature map. Applying global average pooling across spatial and temporal dimensions yields scalar weights for each channel. The weighted average of the channels within the feature map is then computed based on these weights, resulting in the heatmap. This heatmap is then scaled and extrapolated to match the input video size. Finally, each heatmap frame is superimposed onto the corresponding video frame, creating the Grad-CAM frames.

**Fig. 2:**
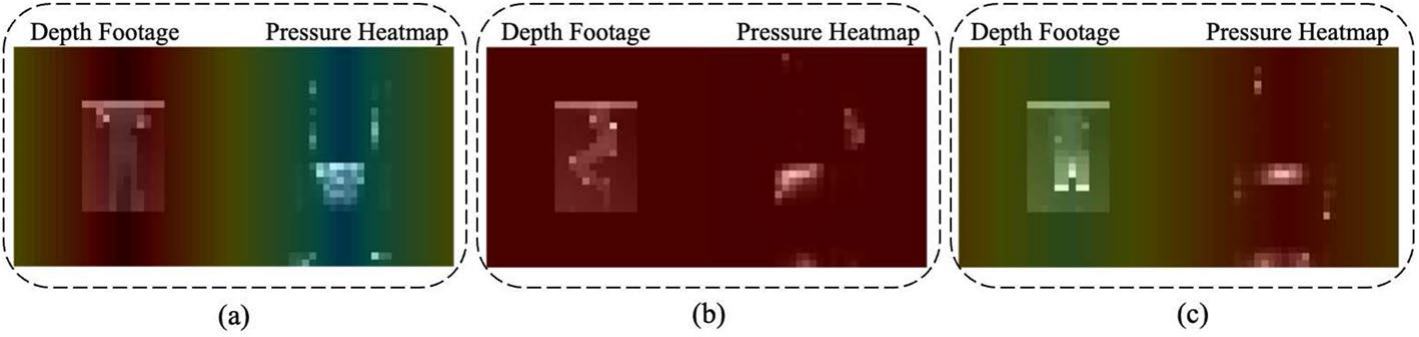
GradCAM visualization for **a** EL, **b** TC, and **c** BG exercises

Figure 2(a-c) displays a sample Grad-CAM frame for EL, TC, and BG exercises, respectively. The more intense red colors represent the areas of the body heatmap that the model was more focused on and considered significant for the prediction. For the EL exercise in Figure 2(a), the model predominantly focused on the depth part of the input to make a prediction. Conversely, in the BG exercise (Figure 2(c)), the model relied more on the pressure data for the prediction. For the TC exercise in Figure 2(b), the model’s attention was distributed across both the depth and pressure parts of the input data.

### Comparison with the state of the art

Table 2 presents a comparison between the findings of previous studies and our own study. We included only the results matching the data type and cross-validation methods used in previous studies.

**Table 2:** Comparison with the state of the art

Model	Cross validation	Data modality	Network architecture	F1 score (%)
[24] and [25]	LOSO	DC-PM	Hybrid Attention Fusion	90.41
	LMSO	DC	2D-CNN	87.2
		PM	1D-CNN-LSTM	74.08
This study	LOSO	DC-PM	I3D	95.74
	LMSO	DC	I3D	90.83
		PM	I3D	75.28

In our comparison with the work of Wijekoon *et al.*, we found that the I3D model outperforms both the 1D-CNN-LSTM and 2D-CNN models used in their study. Wijekoon *et al.* employed a 1D-CNN-LSTM for PM data, where each frame was flattened to form a vector and frames from a time window were appended together to create a single-dimension input feature vector. For DC data, they used a 2D-CNN, where flattened frames within a time window were appended to form a 2D vector [24]. Given that both 1D-CNN and 2D-CNN use flattened data, there is a potential for loss of spatial information. However, the I3D model analyzes the data in both spatial and temporal dimensions, allowing it to effectively capture patterns and dynamics in the exercise sequences. While our approach uses early fusion by concatenating sensor data at the input layer, Wijekoon *et al.* used a late fusion approach with a multimodal hybrid attention fusion architecture [25]. They employed the models previously used in their single modality analysis to independently learn feature representations for each modality, subsequently integrating them through a hybrid attention mechanism. Our early fusion approach, however, demonstrated superior performance.

### Multimodal fine-tuning of the I3D model

In our study, we employed the concept of transfer learning using an Inflated 3D ConvNet (I3D) model which was pretrained on RGB data from ImageNet [36] and Kinetics [37] datasets. This pre-training phase allowed the model to learn a diverse set of features related to color and texture. We then fine-tuned this pre-trained model on depth data, pressure mat data, and a concatenation of both. The depth data, providing information about the distance of objects from the camera, and the pressure mat data, indicating the pressure applied at different points, offered different types of information compared to the RGB data. However, the models were able to adjust the learned features from the RGB data to better fit these new types of data during the fine-tuning process. When depth and pressure inputs were concatenated, the model had access to a richer set of information for making predictions. This approach leveraged the initial understanding of feature extraction from the RGB data, providing the model with a head start and leading to improved performance on the new task.

### Quality of movement vs. classification results

Evaluating our models using data from individuals with disabilities (patients) or older adults (seniors) will influence our classification results. We anticipate a decrease in performance accuracy and increased variability, as our models were originally trained on data from a healthy population and may struggle to generalize effectively to exercises performed with diverse movement qualities. The movement quality among patients and seniors is expected to exhibit lower or more variable characteristics compared to the healthy population, likely attributed to factors such as experiences of pain or limited range of motion. However, this challenge can be mitigated by fine-tuning the model with data specifically from patients and seniors. This approach will enable the models to adapt and generalize to lower movement qualities. We propose that our exercise recognition model, initially trained on healthy population data, serves as a promising starting point for training on patient’s data and initiating the fine-tuning process.

### Generating a quality of movement metric

A similar transfer learning approach can be applied to generate a Quality Of Movement (QOM) metric. If our models have effectively learned feature patterns from the data, they can be used to initiate a transfer learning process for exercise assessment. This may involve transitioning to a binary classification task, distinguishing between correct and incorrect movements, a multi-classification task with categorical labels such as excellent, good, fair, poor, or a regression task to generate a continuous score within the range of 0 to 100. An alternative approach involves combining the exercise recognition model with another model to predict exercise quality scores. For instance, the output of an exercise recognition model (including class probabilities or assigned labels) could serve as a metric to assess the QOMs [23, 38]. The implementation of these approaches needs a comprehensive dataset with well-defined annotations by a trained observer. In the future, we plan to collect data to explore and investigate these approaches further.

## Conclusion

In this study, we present a state-of-the-art 3D-CNN model capable of recognizing lower limb rehabilitation exercises using privacy-preserving depth information and pressure data from an available online dataset. The dataset consisted of a total of 210 videos of 30 healthy individuals performing 7 exercises. We evaluated the effectiveness of this model with three different inputs: depth data, pressure data, and concatenated depth and pressure data. With LOSO cross-validation, the model demonstrated macro F1 scores of 93.80%, 81.45%, and 95.74% for depth data, pressure data, and concatenated data, respectively. Similarly, with LMSO cross-validation, the performance was 90.83%, 75.28%, and 94.77% for depth data, pressure data, and concatenated data, respectively. This outcome highlights the impact of data fusion for accurately classifying the exercises, both in the LOSO and LMSO scenarios. The proposed 3D-CNN model outperforms the previous models as it can analyze data in both spatial and temporal dimensions. Due to its high accuracy, our model is well-suited for recognizing the seven aforementioned exercises in automatic tele-rehab applications. It is essential to recognize that this study focused on a narrow subset of the tele-rehab field. Further research can explore the applicability of our approach to a broader range of exercises.

## Methods

### Proposed approach

We created three exercise recognition models to classify the following: 1—depth videos (DC), 2—sequences of pressure data frames (PM), and 3—concatenated depth videos and pressure frames (DC-PM). We opted for input concatenation, a form of early fusion, for its simplicity and efficiency. It allows us to use a single architecture and access all available information in both data modes simultaneously, potentially to learn features that involve interactions between the modalities. These models were developed to classify all 7 exercises in the dataset. We used a pretrained 3D-CNN model proposed by Carreira *et al.* [39]. This state-of-the-art network, known as “Inflated 3D ConvNets” (I3D), was trained on the Kinetics dataset, which comprises a total of 240,000 training videos of 400 different human actions, including person actions, e.g., drawing; actions involving interactions between individuals and objects, e.g., washing dishes; and actions involving interactions between individuals, e.g., hugging. This model achieved an accuracy of 74.1% when applied to the RGB data from the Kinects dataset. Also, after pretraining on both ImageNet and Kinetics, it demonstrated accuracies of 97.9% and 96.9% when tested on UCF-101 [40] and HMDB-51 [41] datasets, respectively [39]. This architecture has shown promise in multi-modal classification settings [42].

**Fig. 3:**
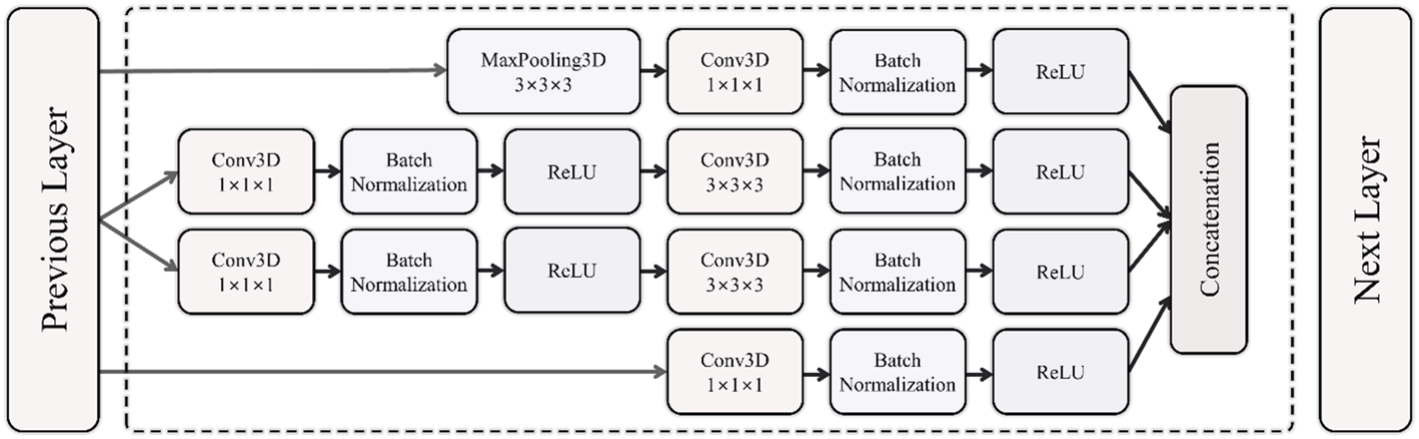
Schematic representation of the 3D Inception module. The activation function, Rectified Linear Unit (ReLU), introduces non-linearity to the model. This non-linearity makes the model capable of learning complex spatio-temporal patterns from the video data, in light of the presence of 3D convolutions

The I3D model uses 3D convolution to learn spatiotemporal information directly from input videos [43]. More specifically, the architecture consists of a series of 3D Inception modules followed by 3D max pooling and batch normalization layers. The Inception module, as depicted in Figure 3, operates with parallel 1$\times$1$\times$1 and 3$\times$3$\times$3 3D convolution kernels and a 3$\times$3$\times$3 max pooling operation using the same input data, merging their outputs into a single output. Incorporating 1$\times$1$\times$1 convolution layers reduces the dimensions of the input data within the network and, therefore, reduces the computational cost. The 3$\times$3$\times$3 convolution layer enables the network to learn spatiotemporal features at a different scale. The dimensions of the input data are reduced by the 3$\times$3$\times$3 max-pooling layer while allowing the extraction of different features simultaneously. Max-pooling is thus employed to extract more features from the input data [44]. A dropout layer was also used to prevent overfitting of the models.

The input for the 3D models consists of videos with a size of N $\times$ R $\times$ C $\times$ 3, where N represents the number of frames in the video. Each frame has a resolution of R $\times$ C $\times$ 3, where R and C are the number of rows and columns, respectively. Also, 3 indicates the number of channels.

**Fig. 4:**
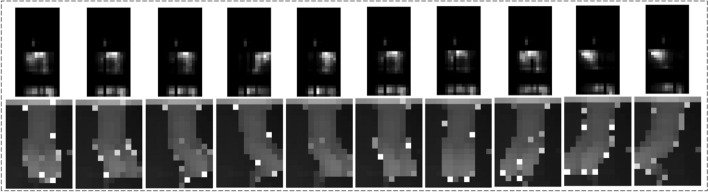
Visualization of pressure and depth data (subject #1, knee-rolling exercise)

For preprocessing, each depth video was downsampled to 158 frames, which is the shortest length of depth videos in the dataset. Each frame was zero-padded to 32$\times$32 pixels. Likewise, the pressure videos were downsampled to 252 frames, and each frame was zero-padded to 32×32 pixels. To create the concatenated input video, the pressure videos were also downsampled to 158 frames to be consistent with the depth data. Corresponding depth and pressure frames were zero-padded and concatenated next to each other to form 32 × 64 input frames. An example of input videos for PM (top frames) and DC of knee-rolling exercise is depicted in Figure 4.

To classify the performed exercises, we used categorical cross entropy as the loss function in our classification task. The categorical cross-entropy loss function is defined as follows:

4 $$\begin{aligned} f(x) = -\frac{1}{N}\sum _{i=1}^{N}\sum _{c=1}^{C}1_{y_{i}\in C_{c}}\text {log}(p_{model}[y_{i}\in C_{c}]). \end{aligned}$$

The summation is performed over *N* observations (the training sample size), where *i* iterates over the observations and *c* iterates over the number of classes (exercises). The term $1_{y_{i}\in C_{c}}$ represents an indicator function that equals 1 if the $i^{th}$ observation belongs to the $c^{th}$ category and 0 otherwise. The logarithm of the predicted probability by the model for the $i^{th}$ observation belonging to the $c^{th}$ category is calculated. The objective was to minimize this loss function during the training phase. To optimize the model, we employed the Adam optimizer [45]. The Adam optimizer adapts the learning rate for each weight of the neural network using estimations of the first moment and the second moment of the gradient. This adaptive learning rate scheme aids in effectively updating the weights during the training process.

### Dataset

This study used depth video recordings and body pressure data from the dataset published by Wijekoon *et al.* [24]. The data were collected from 30 healthy participants, comprising 18 females and 12 males. Fourteen subjects were aged 18 to 24, while the rest of the individuals were aged 24 to 54. It is noteworthy that 8 participants had some background in physiotherapy, either as physiotherapists or physiotherapy students, thus having a good knowledge of the exercises. The participants performed the 7 different lower-limb rehabilitation exercises listed in Table 3. These exercises are frequently recommended by clinicians for the prevention or management of musculoskeletal pain [24].

**Table 3:** List of exercises in the MEx dataset [24]

Exercise	Starting Position	Action
Knee-rolling (KR)	Lying on back, knees bent	Roll knees side to side, keeping upper body still
Bridging (BG)	Lying on back, knees bent	Lift hips off floor, hold for 5 seconds, and lower
Pelvic tilt (PT)	Lying on back, knees bent	Tighten stomach muscles, press lower back to floor, rise bottom, hold for 5 seconds, relax
The Clam (TC)	Lying on side, knees bent	Rotate leg and open knee while keeping hips aligned, return to starting position
Repeated Extension in Lying (EL)	Lying face down, palms on floor	Straighten elbows, push upper body up for 2 seconds, and lower back down
Prone punches (PP)	On all 4s	Punch arms forward while keeping the core stable
Superman (SM)	On all 4s	Extend the opposite arm and leg for 5 seconds while keeping the core stable

The participants performed all exercises while lying down on the pressure mat. A depth camera on top of the participants recorded their body movements from an aerial perspective. To ensure alignment, the top of the depth frames matched the top of the pressure mat. As a result, body parts above the shoulders were not visible to the depth camera. The participants were asked to perform each exercise for 60 seconds without any instruction. This approach aimed to mimic a natural setting where participants, acting as patients, performed the exercises independently at home without the guidance of a physiotherapist [24].

**Fig. 5:**
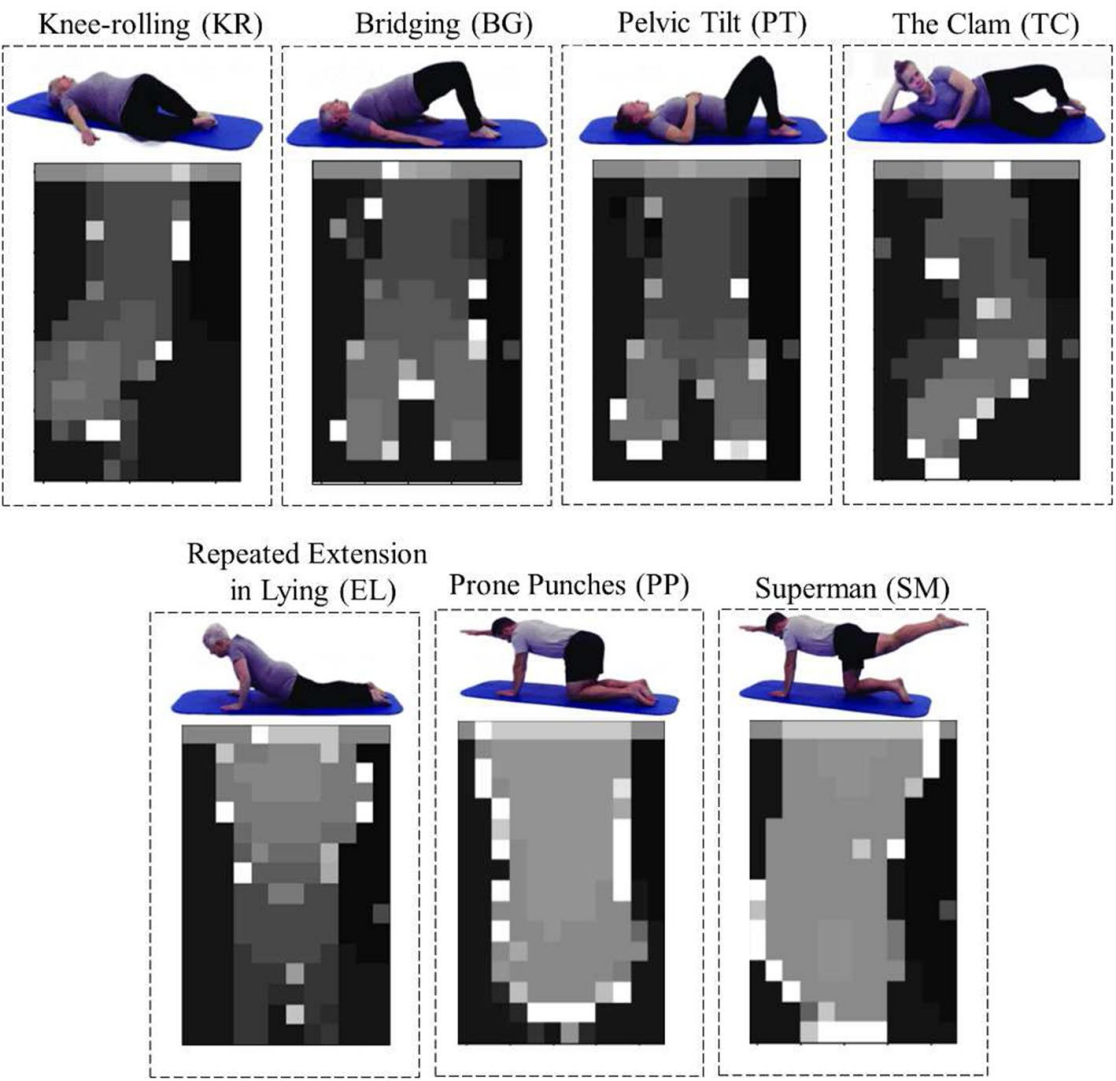
Seven exercises performed by Subject #1 and their corresponding depth frames, adapted with permission from [25]

**Fig. 6:**
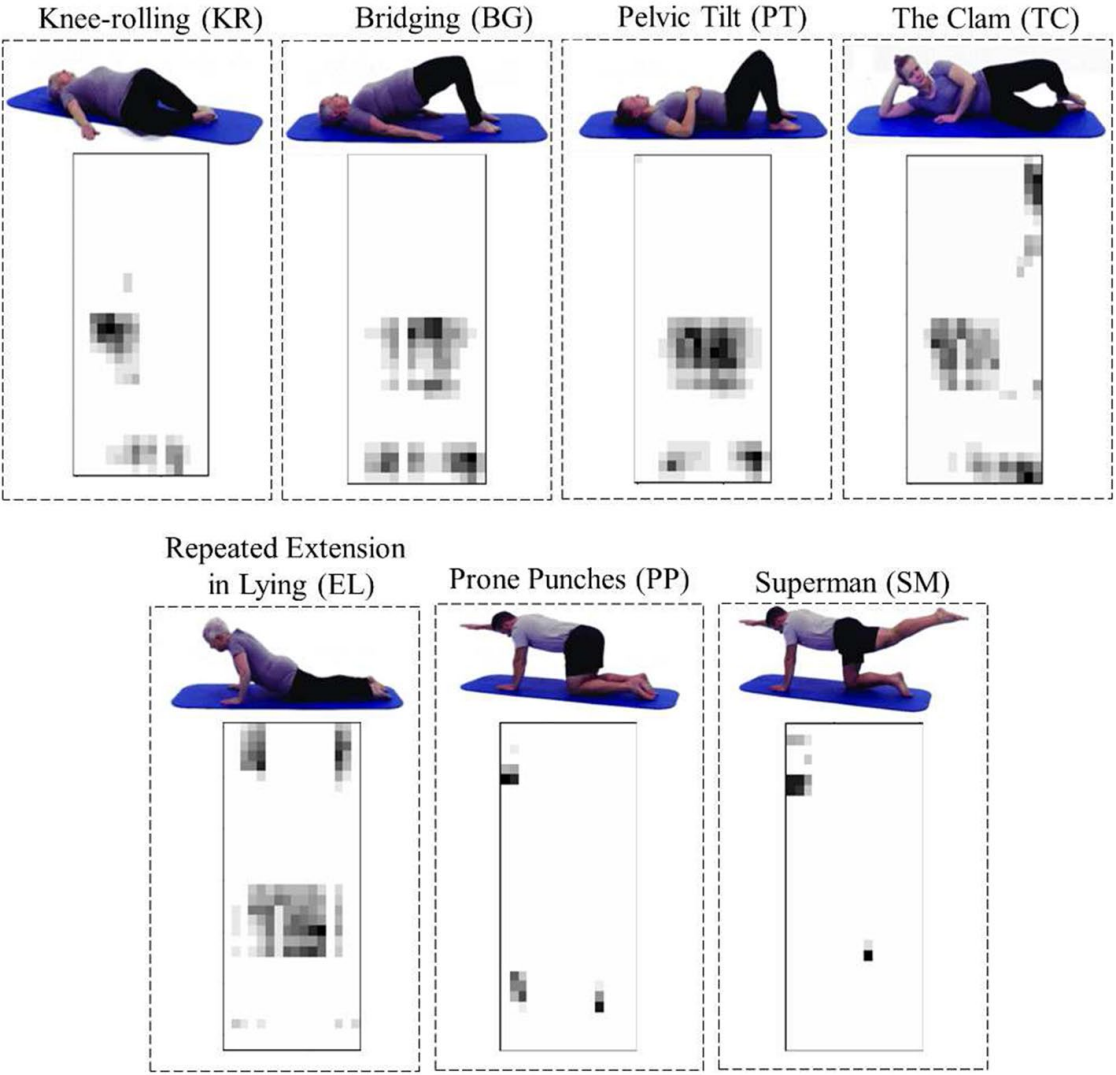
Seven exercises performed by Subject #1 and their corresponding pressure frames, adapted with permission from [25]

The depth data were captured by an Obbrec Astra depth camera at 15 Hz with a resolution of 320×240 resized to 12×16. The pressure data were captured by a SensingTex pressure mat at 15 Hz with a resolution of 32×16. The depth and pressure data were simultaneously recorded from each participant. Overall, we had 210 (30 participant × 7 exercises) single-channel grayscale videos from each data source. The data values in each video were normalized using min–max scaling, ranging between 0 and 1. Some examples of depth and pressure frames from subject #1 for all 7 exercises are depicted in Figure 5 and Figure 6, respectively [24].

## Data Availability

The datasets generated and/or analysed during the current study are available in the UCI Machine Learning repository, https://archive.ics.uci.edu/dataset/500/mex
